# Towards quality management of artificial intelligence systems for medical applications

**DOI:** 10.1016/j.zemedi.2024.02.001

**Published:** 2024-02-27

**Authors:** Lorenzo Mercolli, Axel Rominger, Kuangyu Shi

**Affiliations:** Department of Nuclear Medicine, Inselspital, Bern University Hospital, University of Bern, Freiburgstrasse 18, CH-3010 Bern, Switzerland

**Keywords:** Artificial intelligence, Machine learning, Quality management, Risk analysis

## Abstract

The use of artificial intelligence systems in clinical routine is still hampered by the necessity of a medical device certification and/or by the difficulty of implementing these systems in a clinic’s quality management system. In this context, the key questions for a user are how to ensure robust model predictions and how to appraise the quality of a model’s results on a regular basis.

In this paper we discuss some conceptual foundation for a clinical implementation of a machine learning system and argue that both vendors and users should take certain responsibilities, as is already common practice for high-risk medical equipment.

We propose the methodology from AAPM Task Group 100 report No. 283 as a conceptual framework for developing risk-driven a quality management program for a clinical process that encompasses a machine learning system. This is illustrated with an example of a clinical workflow. Our analysis shows how the risk evaluation in this framework can accommodate artificial intelligence based systems independently of their robustness evaluation or the user’s in–house expertise. In particular, we highlight how the degree of interpretability of a machine learning system can be systematically accounted for within the risk evaluation and in the development of a quality management system.

## 1 Introduction

The availability of computational power and of large amounts of data made it possible for artificial intelligence (AI) to become one of the most rapidly developing field of science and technology over the last two decades. The potential of AI in healthcare was quickly recognized and research has been directed first to medical domains where large amounts of more or less standardized data are generated. This includes disciplines that work with image data, such as radiology, nuclear medicine, radiation oncology or pathology (see e.g. Refs. [1], [2], [3], [4]). The benefits of AI systems in medicine, in particular medical imaging, are manifold and span different areas and modalities.

While research on AI applications in medicine has grown rapidly, the use of such tools in clinical routine has not yet become standard practice. Like every other software, also AI tools have to fit into a well-defined and potentially rather complex clinical process. Furthermore, the software usually has to be classified as a medical device and therefore needs to satisfy high standards of robustness and reproducibility. While a growing number of CE marked or FDA approved AI software tools become available on the marked (see e.g.https://grand-challenge.org/aiforradiology/), the certification procedures are still part an ongoing discussion, as can be seen e.g. in Refs. [5], [6], [7], [8].

Of course, a risk assessment as well as quality management (QM) of AI tools is a key requirement for bringing the potential benefits of AI into clinical routine. The aim of this paper is to propose a conceptual framework for QM of AI tools in clinical processes and to hint towards the elaboration of robust clinical workflows that include AI.

We argue that the AAPM Task Group 100 methodology, described in the report No. 283 [9], provides a convenient framework to develop a risk-driven QM system for a clinical workflow that contains AI. This was developed for complex and high-risk clinical processes in radiation oncology. With a simple example of a generic imaging workflow, we show how to develop a QM program for AI. Furthermore, we discuss how methods from adversarial attacks/defenses and interpretable AI can be taken into account in a systematic way.

## 2 Conceptual considerations for QM of AI

The importance of robustness of AI for medical applications, in the sense of coping with errors or faulty input, cannot be overstated. It is key for a widespread adoption and integration of this technology, as can be seen from the vast literature on the topic (see e.g. Refs. [10], [11], [12] and references therein). However, it is often very difficult to assess the robustness of an AI tool. Nevertheless, we should to find a way to deal with the potential failures of AI systems in clinical practice in order not to hamper their clinical implementation. We believe that there are two essential apsects. Firstly, there is a shared responsibility between the vendor and the user with respect to the robustness of the AI tool. Secondly, the robustness requirement should not be applied to a software alone but rather to the whole clinical process.

The standard practices of radiation oncology, and in particular to proton therapy, provide useful guiding principle for our argument: a particle accelerator with the corresponding beam line and treatment head is an infinitely complex device with almost uncountable individual components that may fail at any time. Often proton therapy facilities are unique prototypes built in research centers with strong ties to accelerator and high-energy physics research institutes (see e.g.  https://www.psi.ch/en/protontherapy) and it is therefore not unusual to employ non-certified equipment or software in a clinical process that encompasses high risks for patient harm. In such cases, the clinic takes the full responsibility for the device’s risk management and QA. We believe that the clinical implementation of AI can follow this blueprint and learn from the experiences in this field.

### 2.1 Threat model for medical devices

In general, stringent national and international regulations assign the responsibility for the correct functioning of a certified medical device to the vendors. Depending on the medical devices’ classification, i.e. the associated risks, notified bodies perform conformity assessments. As seen e.g. in Ref. [5] or in the European Union’s draft of the Artificial Intelligence Act, regulators are taking actions in order to provide a regulatory framework for AI applications in the medical domain. Of course, the robustness of an AI system needs to be addressed within such a framework.

Medical device certification requires an in-depth risk analysis by the vendor. For AI products, this is particularly challenging. Some scholars have therefore put forward the necessity of model interpretability for the clinical use of AI (see e.g. Refs. [13], [14], [15], [16], [17], [18], [19]). However, while interpretability can certainly aid the risk assessment and increase the user’s trust of an AI tool, in our opinion it is too restrictive to strictly require every medial application of AI to be interpretable. Also, the degree of interpretability and the deduction of robustness measures therefrom can be quite subjective (see e.g. Ref. [19] for a review of and future challenges of interpretability in healthcare).

It was found to be rather simple to construct an input for a trained AI model that causes erratic predictions [20] (see also the reviews [21], [22], [23]). Such an input is called *adversarial example*. This discovery has raised lots of concerns about AI’s robustness. The basic idea behind adversarial examples is to design small perturbation to the model input, which are hardly perceivable to humans and which will cause the AI model to make a wrong prediction. Illustrative examples can be found in Refs. [24], [25] and on http://www.cleverhans.io. Adversarial examples can be thought of the AI’s equivalent to optical illusions for humans. Interestingly, even the methods from interpretable AI can be vulnerable to adversarial examples, as shown in Ref. [26].

In response to the challenges posed by adversarial examples, the authors of Ref. [27] formulated guiding principles on how to evaluate the robustness for a model and even provided a checklist for model developers. A basic requirement is the definition of a *threat model*, i.e. the rules and restriction under which to assess the robustness. A threat model for an AI system should, in our opinion, be part of a medical device’s scope definition. With a precise definition of the threat model, the robustness of a model is well-defined and in some cases even computable, as discussed in Refs. [28], [29]. The definition of a threat model is also helpful for mitigating the lack of robustness. A threat model should encompass the goals (e.g. simple misclassification, targeted attack, etc.), knowledge (e.g. white or black box attack, access to data sets, etc.) and capabilities (size and type of input perturbations, etc.) under which the robustness of an AI system is assessed.

While adversarial examples have shown the limits of out-of-sample variance as a measure for a model’s robustness and generalization capabilities, they allow to test and assess the robustness of an AI system under extreme and/or worst case. As pointed out in Refs. [27], [30], evaluating adversarial robustness of a model can provide some useful insights about the behavior of the AI model. For risk assessments of AI systems, adversarial examples can therefore be an invaluable tool.

We advocate for vendors of a clinical AI system to provide a detailed documentation about the robustness assessment and risk evaluation of their system. This should include in particular specifications about the employed threat model as well as robustness tests and measures. It would be highly desirable that the users of an AI software could have insight in the risk evaluations of the CE markings or FDA approvals. The shared responsibility between the vendors and users means that this kind information needs to be shared. Otherwise the user has to assume the worst possible risk for the AI software.

### 2.2 Quality management program for AI users

In the previous section we discussed the vendors/developers responsibilities with respect to robustness of an AI software. The discussion was purely focused on the software tool itself. We strongly believe that the whole clinical process needs to be robust in order to provide the necessary trust to the user. Focusing on a software tool alone might hamper the clinical implementation and delay the exploit the advantages of AI.

Clinics usually have their workflows mapped in a QM system that is closely related to the clinic’s risk management. Every software tool that is used in a clinical workflow is therefore part of a clinic’s risk analysis and QM program (as emphasized also in Ref. [31]). Often devices and software are not explicitly listed or considered in a clinic’s risk assessment, because there is sufficient trust in the medical device certification. If we want to use an AI system in a clinical workflow, its risks need to be assessed and QA measures need to be defined.

From the user’s perspective, we think that AI tools should be approached in the same way as an external beam radiotherapy facility regarding risk assessment and QA. Radiation oncology has shown how unreliable and very complex systems can fit in a clinical workflow that ensures a very high level of patient safety.

The technical QA program in radiation oncology clinics is traditionally based on national and international recommendations. Such recommendations usually focus on assessing functional performance measures of a device and rarely take into account the whole clinical workflow. In particular in radiation oncology, faulty outcomes are often due to issues related to the workflow and not necessarily because of technical failures. Furthermore, these recommendations often have problems to keep up with the rapid technological advances due to the rather lengthy publishing process. This is why the TG-100 of the AAPM put forward a risk-based methodology that directs the QA measures in a resource-efficient way, while providing an optimal patient safety (see e.g. Section 3.B. of Ref. [9]). Since it considers a specific clinical workflow as a whole, rather than just functional performance measures, it allows for a swift implementation of new technologies by the user.

Using the wording of Section 2.1, the threat model for a full clinical workflow should not only cover failures of individual components of the process but the process as a whole. This is one of the strong points of the proposed methodology, as the QM measures are conceived based on the risks of individual steps in the whole workflow.

### 2.3 AAPM TG-100 methodology for AI

The AAPM TG-100 methodology relies on three principles for risk assessment and mitigation^1^: process mapping, failure mode and effect analysis (FMEA) and fault trees (FT). The methodology relies on an iterative procedure: depending on the outcome of the first risk assessment, the process map, FMEA, FT and QM program can be adapted and the risk assessment is repeated until the clinical workflow under consideration has an acceptable risk of hazards.

The process for risk analysis and mitigation outlined in Sec. 5 of Ref. [9] involves the following steps.

#### 2.3.1 Process mapping

In order to assess the risk of a process, it is useful to start with a graphical representation of the whole process. The level of detail of the process map should be considered carefully as it will directly impact the risk analysis. The process map emphasizes the fact that the whole clinical process is under consideration.

#### 2.3.2 Failure mode and effect analysis

“FMEA assesses the likelihood of failures in each step of a process and considers their impact on the final process outcome.” Sec. 5.B. [9]. This is sometimes also referred to as a bottom-up approach since the analysis starts from possible failures in the process steps.

For every step in the process map, the risk of failure and its consequences are evaluated. As discussed in detail in Section 4.D. of Ref. [9], the FMEA is a prospective risk assessment, i.e. the risk quantification is based on expert knowledge.

In a first iteration, the FMEA does not consider any previous QA measures that might already be in place in order not to introduce any bias. Since FMEA is a bottom-up approach, we start by identifying as much failure modes, i.e. ways that each step in our workflow could fail, as possible. Then, the causes and the impact on the final outcome of each failure mode must be determined. Each failure mode is quantified with three figures of merit: occurrence *O*, severity *S* and lack of detectability *D*. Therefrom, the *risk priority number RPN* is computed as

(1) RPN=O·S·D

The determination of the values for O,S, and *D* is often challenging. Usually, it is strongly advised to elaborate the quantification of the FMEA in a cross-professional team. The values of O,S, and *D* range from 1 to 10 and correspond to the definition in Tab. II of Ref. [9]. They span the following ranges.

• Occurrence *O*: goes from O=1 for “failure unlikely” (frequency <0.01%) to O=10, which is defined as “failures inevitable” (frequency >5%).
• Severity *S*: ranges from S=1 for “no effect” to S=10 meaning a “catastrophic” event.
• Lack of detectability *D*: quantifies the likelihood that a failure in a process step is not detected. The value spans D=1, i.e. an error is detected with a probability ⩾0.99%, to D=10 for failures that are detected ⩽20% of the cases.

#### 2.3.3 Fault tree analysis

A FT is a useful tool to visualize how failures propagate through the process. The FT analysis complements the FMEA and helps to uncover risks, and in particular interconnection between process steps, that might be somewhat hidden in the FMEA. One starts with a failure in the process outcome and then identifies all possible hazards that could possibly lead to this failure. The FT analysis starts with the error at the end of the process, e.g. harm to the patient or personnel. Then one has to find all possible sources in the workflow that might lead to this hazard. It depends on whether there are multiple factors that need to be satisfied in order to produce a failure (logical “and” gate) or if a single factor can lead itself to an error (logical “or” gate).

#### 2.3.4 QM program

Once the risk of the process is assessed with a FMEA and FT, the QM program is set up to mitigate the major risks that were identified in the FMEA and FT. These risks are reassessed after mitigation strategies are in place, i.e. the FMEA’s *RPN* values guide the users to adapt clinical workflow as to decrease the O,S and *D* values to an acceptable risk figure. To this end, also the process map and the FT might require a revision. In the end, the FT will indicate the process steps that require most QA measures. The AAPM TG-100 methodology therefore allows to allocate the resources for QA where they truly matter in terms of risk and its mitigation.

## 3 Example for a clinical implementation of an AI tool

Let us see how we can apply the AAPM TG-100 methodology to a clinical workflow that includes an AI system. The example at hand is kept as simple as possible and focuses on the AI related parts of the workflow.

### 3.1 Process mapping

**Fig. 1:**
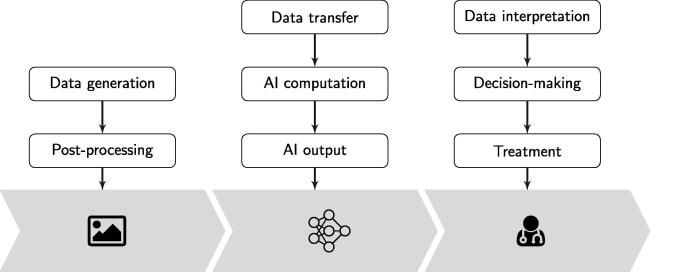
Simplified clinical process with AI support. As a concrete example, one can think of this as a PET/CT image of an oncological patient where the lesion detection is done with AI.

Fig. 1 shows a process map, which is the first step towards our risk assessment and QA program design. Our example can be thought of as a generic version of an imaging workflow. First, we generate and post-process the data. This could be e.g. performing a PET/CT scan and the reconstruction of the image. Of course, we omit several steps of a real clinical workflow, such as e.g. patient referral or the safe operation of a device.

The data is then transferred to an AI system. In real life, this step might require quite some attention. We ignore issues related to the actual data transfer, data format, integrity checks, etc. The main task in this step is that the AI model computes a certain output. Staying with the example of a PET/CT, the AI system could be a model that automatically segments organs or detects lesions.

In our clinical workflow, we do not allow the AI system to take direct action on the patient or the treatment. The output from the model is interpreted by a physician or an interdisciplinary board of physicians, who in turn will decide on the further procedures. The AI system should therefore be thought of as a decision support system.

### 3.2 Failure mode and effect analysis

In Table 1 we provide a simplified FMEA for the process shown in Fig. 1. Table 1 shows that an AI system fits very in a FMEA. It is considered simply as a subsystem and/or step in the clinical workflow that takes some input from the previous subprocesses and produces some output that is needed in the subsequent steps. Of course, there are many things that can go wrong in an AI pipeline. We condensed the causes of a faulty model prediction to a *hardware failure*, *unstable model* and *improper input*.

**Table 1:** FMEA risk quantification for the second and third step in the processes of Fig. 1.

Failure mode	Cause	Effects	*O*	*S*	*D*	*RPN*
AI system.						
Faulty data transfer	• Network failure	No or faulty data	4	4	1	16
	• Wrong data format		1	6	1	6
Faulty model prediction	• Hardware failure	Faulty AI output	1	5	1	5
	• Non-robust model		3	8	10	240
	• improper input		2	5	3	30

Interpretation and decision-making.						
Faulty data interpretation	• Human error	Patient damage	3	10	4	120
	• Suboptimal reading conditions		2	10	2	40
	• Faulty AI output		6	10	2	120
Wrong treatment decision	• Insufficient decision support	Patient damage	3	10	4	120
	• Miscommunication		2	10	1	20
Wrong treatment	• Faulty prescription	Patient damage	2	10	2	40
	• Faulty treatment application		2	10	1	20

Nowadays, a hardware failure is fairly rare and the chance to pass undetected is minimal. The consequences might be, however, somewhat severe (e.g. a major delay in the treatment). This is why we assigned the values O=1,S=5 and D=1 which gives a rather low value of RPN=5.

Without knowing any details about the AI model’s robustness, medical device certification or interpretability, we have to assume that it is inherently vulnerable and unreliable. The occurrence might still be limited with O=3, i.e. we do not expect a maleficent adversarial attacks and assume that the input data corresponds to what the model expects. However, the severeness can be high as faulty output data might lead to wrong treatment decisions and possibly without being detected. Imagine e.g. that tumor lesions in a PET/CT image are not detected by the AI system. Therefore, we need to assign a high severity of S=8 and a low probability to detect the error D=10.

Finally, a faulty model output might be produced because the input data is outside the model’s validity. Assuming that the data pipeline is more or less robust, e.g. the model will not receive an MR image when it expects PET/CT data, the occurrence should be as low as O=2. The severity might be higher S=5, but we expect the model to produce an output that is more or less easily detected as faulty, hence D=3.

### 3.3 Fault tree

**Fig. 2:**
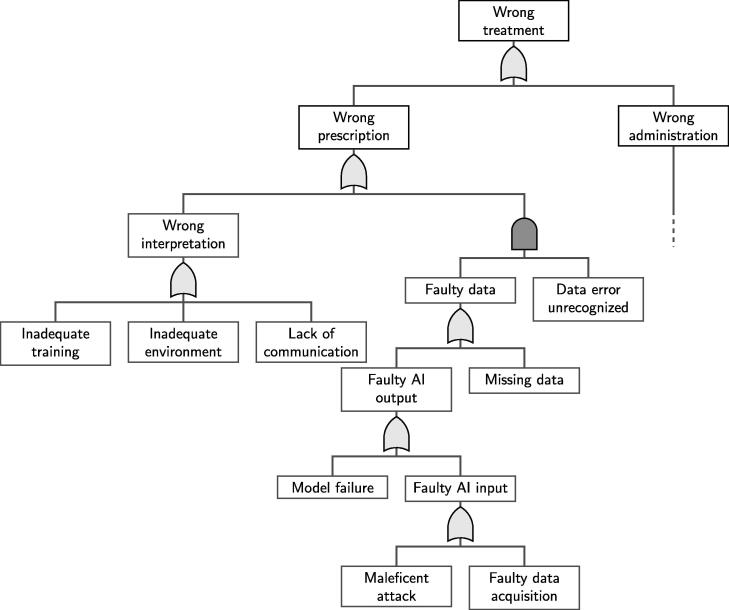
Simplified FT for the process of Fig. 1. The symbol  represents an “and” gate while  is an “or” gate.

In Fig. 2 we show a FT for the process in Fig. 1, but for simplicity’s sake we omitted the continuation of certain branches in the FT. As seen in Fig. 2, the wrong treatment can be caused either by a wrong prescription or by a wrong administration of the treatment. It is then clear that *and* gates represent a safety feature, since multiple conditions must be met in order to produce a failure. On the other hand, *or* gates should be investigated more closely since they bear the risk of error propagation. Therefore, any QA measure should start at an *or* gates.

The AI related branch of the FT is fairly simple. There are two possibilities that can produce a wrong or incomplete model output: either there is a problem with the input or with the model itself. The input could be faulty or incomplete e.g. because of random variations in the data acquisition, wrong data format, wrong data protocol, incomplete data transfer, adversarial attacks etc. Regarding a failure of the model, imagine that the model is not adequate for the task or that the model performance is not as expected.

### 3.4 QA program

Based on the process map, the FMEA and the FT we can now design the risk mitigation measures and the QA program for our clinical workflow. The FMEA and the risk quantification show which steps are most in need of risk mitigation measures. On the other hand, the FT give some insights in where the propagation of errors is most efficiently blocked. If a single QM step or measure is sufficient to block an error from creating an incident depends on the specific case. In general, however, it is advisable to have multiple *and* gates in the FT preventing the propagation of errors. Having multiple QM measures that can block an error will certainly reduce the value of *RPN*, mostly because of a decreased *D* value.

Considering our FMEA, it is clear that we should focus our QM efforts on the AI system’s lack of robustness, the data interpretation, the decision-making process and the problems related to contraindication (see Table 1). In practice this would mean an increased attention to the clinician’s training, a four-eye sign-off procedure for the decision-making, allocation of sufficient time for the data interpretation or similar measure. From the FT, we know that faults in the AI output could be compensated by a robust data interpretation. In a second iteration of the analysis, we could therefore lower the value of *D* in the FMEA if our QA measures in the decision-making and prescription process can prevent a faulty AI output to propagate further in the FT. This example illustrates nicely how even an unreliable AI system can be implemented in a robust and safe clinical workflow.

### 3.5 Risk mitigation for AI systems

Basically there are two strategies that users can peruse: risk mitigation through the clinical process or through the robustness of the AI tool itself. In Section 3.3 we saw how the data interpretation and decision-making process can make up for the AI system’s lack of robustness. In the FMEA this would be expressed in a low value of *D* and possibly *S*.

However, the user might wish to reduce not only *D* or *S* but also *O*. As discussed in Section 2.1, the medical device certification involves a risk analysis. The user might choose to rely on this risk assessment. However, given that in general the risk assessments of medical devices are not publicly available (and often not even checked by the notified bodies), the user should have the knowledge and resources to understand the risks of the AI tool before assigning low *O* scores in the FMEA.

In analogy to the recurring dosimetric measurements of a QA program in radiation oncology, we believe that setting up a series of periodic in–house and vendor independent tests of the AI system’s performance can provide confidence and trust in the AI model. This could be e.g. a user-specific test data set that includes adversarial examples, randomly generated data, corrupted or otherwise faulty input. It is important to keep in mind that such an in–house test should cover as much as possible the intended use of the software, as defined e.g. in the CE labeling or FDA approval of the software.

Another important aspect for increasing the robustness of an AI model is its interpretability and/or explainability (see e.g. Refs.[33], [34]). The basic idea of interpretable AI is to find models and/or develop methods that allow for a human interpretation or explanation of the model’s output. Some scholars have recently argued that interpretability should be a requirement for AI systems in medical applications (see Refs. [14], [15], [16], [35]). We are convinced that interpretability can play a major role in assessing the possible risks of a AI model and thereby lower the values of *O* and *S* in the FMEA. Note, however, that interpretation of a AI model might require significant expertise and the authors of Ref. [16] showed how current interpretation/explanation methods might fail at providing decision support (see also Refs. [36], [19]).

## 4 Discussion

In the previous sections we illustrated the importance of performing risk assessments and QA measures focusing on the full clinical process rather than just on individual tools or process steps. It is therefore the clinic’s responsibility to perform such a risk evaluation. To this end, the AAPM TG-100 methodology provides a conceptual framework which can accommodate easily AI tools. Of course, data based assessments are preferable but reliable data on failure probabilities, severity, etc. is often not available. Depending on the user’s in–house knowledge, the risk evaluation for the AI part of the clinical process can be split into the following categories.

• The AI software is a certified medical device. This means that there is a risk evaluation of the software, depending on the type and intended use of the medical device. The user can assign a low score for *O* in the FMEA. If the user does not trust the vendor to adhere to the best practices or is otherwise sceptical of the AI tool’s robustness (as e.g. also Ref. [37] suggests), the procedure discussed in Section 2.3 is still applicable under the circumstances described in the following tow points.
• The user has the means and expertise to asses the AI software’s robustness and can therefrom deduct the risk scores for the FMEA. While this might be the case in larger clinics, smaller centers will likely need to be conservative and apply the next category.
• The AI software is a black box and the user has no means to assess the software’s robustness. If so, the user should allocate high *RPN* numbers to the AI tool. The mitigation strategy and QM should then focus on the other steps in the clinical process.

It is apparent that the AAPM TG-100 methodology is agnostic towards interpretability of the AI tool and the trust that we put into it. Implementing an interpretable AI model can alter the risk assessment, i.e. the *RPN*, and establish confidence in the model’s performance. The same applies to in–house test data sets and best practices when constructing the model. The key advantage of the AAPM TG-100 methodology is that it does not matter how well a model is interpretable or how robust it is. It is always possible to implement it in a clinical workflow. The whole workflow and the QM measures will then simply be adapted according to the risk that the AI model represents.

In case of high risk scores for the AI model in the FMEA, the clinical process needs to mitigate the risk of hazards. In the example of Section 3, the key component is the *and* gate in the FT in Fig. 2 that connects the faulty output from the AI model with the event that the error is not recognized by the physicians in the decision-making process. Hence, a wrong prescription will only occur if the AI output is incorrect and the error remains unrecognized in the decision-making process. Due to this *and* gate, we could in principle focus all our QA measures on the decision-making process and ensure that every AI output error gets recognized and we can compensate for the high *RPN* of the AI system in the FMEA (see Table 1a). Or in other words, in a second iteration of the FMEA we would reduce the value of *D* due to the “and” gate in our FT.

Note well that if AI were to take direct and/or automatic action on the patient treatment, the methodology would not change and such a process would still fit in the AAPM TG-100 framework. The FMEA would feature high *S* and *D* values and QM measures would have to be directed to lowering these figures to an acceptable level.

It is important to be aware of the limitations risk assessment, as discussed in Section 2. On one hand, if the clinical workflow under consideration is complex a full FMEA can be time-consuming (see e.g. Sec. 6.4 in Ref. [32]). This is particularly true in situations where the quantification is done by a cross-professional team. The quantification of a risk in terms of O,S and *D* is subjective, since reliable empirical data is often missing. This makes the *RPN* a rather uncertain figure of merit with possibly large variations. Furthermore, the *RPN* might not reflect the true risk (is it meaningful to simply multiply O,S and *D*?) and there are issues related to the distribution of the numerical values (see Ref. [38]).

Also the FT analysis has some limitations. E.g. it can become difficult to visualize and account for complex interactions between the levels of the FT.

## 5 Conclusions

Despite the huge potential benefits that AI can bring to healthcare, there are some fundamental issues. One of the mayor concerns for the clinical implementation of AI is the robustness of such tools. In this paper, we propose a conceptual framework and discuss how an AI system can be implemented in a clinical workflow.

The possible robustness issues of AI require a conceptual framework that can address the risks systematically. We argue that AI in healthcare should draw from the field of radiation oncology, where complex and possibly unreliable equipment that can cause high patient damage is being used in clinical routine. First, we take the view that there is a shared responsibility between the vendors and users when implementing AI system in a clinical workflow. On one hand, the vendors (or developers) should adhere to best practices and, most importantly, should disclose how they address the robustness of their systems. On the other hand, the users are responsible for implementing the AI system in a QM system and setting up appropriate QA measures. For both, the assessment of a model’s robustness is crucial and adversarial examples can play a key role in tackling these questions.

We advocate to use the methodology from the AAPM Task Group 100 report [9] develop a QA program for complex clinical processes that include AI systems. With a generic example of an imaging workflow we illustrate this point by performing a process map, FMEA and FTA. One of the key points of the AAPM Task Group 100 [9] framework is that the whole clinical process is considered in the risk assessment and therefore allows for an efficient construction of a QA program. Also, this methodology does not depend on reliability of an AI system. Rather, it provides a framework that can accommodate any level of AI robustness or user’s expertise to evaluate it. Of course, the risk evaluation will change according to a model’s robustness and therefore also the necessary QA measures adapt. We stress that interpretability of a AI model is not a strict requirement in this framework, but becomes a risk mitigation strategy that can significantly reduce the risk scores in the FMEA.

## Data Availability Statement

The code used to extract the data is distributed by the authors as open-source. The patient data cannot be made available on request due to privacy/ethical restrictions.

## Declaration of Competing Interests

The authors declare that they have no known competing financial interests or personal relationships that could have appeared to influence the work reported in this paper.

## Footnotes

1 Note that this methodology is in line with the recommendations of the Particle Therapy Co-Operative Group, where a combination of top-down and bottom-up risk assessments is viewed as most effectively (see e.g. Section Section 6.1 in Ref. [32]).
